# Non-random distribution of microsatellite motifs and (TTAGGG)n repeats in the monkey frog *Pithecopus rusticus* (Anura, Phyllomedusidae) karyotype

**DOI:** 10.1590/1678-4685-GMB-2019-0151

**Published:** 2020-01-13

**Authors:** Julia R. Ernetti, Camilla B. Gazolla, Shirlei M. Recco-Pimentel, Elaine M. Luca, Daniel P. Bruschi

**Affiliations:** 1 Programa de Pós-graduação em Ciências Ambientais, Área de Ciências Exatas e Ambientais, Universidade Comunitária da Região de Chapecó, Chapecó, SC, Brazil.; 2 Programa de Pós-graduação em Genética, Departamento de Genética, Universidade Federal do Paraná, Curitiba, PR, Brazil.; 3 Departamento de Biologia Estrutural e Funcional, Universidade Estadual de Campinas, Campinas, SP, Brazil.; 4 Departamento de Zootecnia e Ciências Biológicas, Universidade Federal de Santa Maria, Campus de Palmeira das Missões, Palmeira das Missões, RS, Brazil.

**Keywords:** Amphibia, Fluorescence *in situ* Hybridization, repetitive DNA

## Abstract

The monkey frog, *Pithecopus rusticus* (Anura, Phyllomedusidae) is endemic to the grasslands of the Araucarias Plateau, southern Brazil. This species is known only from a small population found at the type locality. Here, we analyzed for the first time the chromosomal organization of the repetitive sequences, including seven microsatellite repeats and telomeric sequences (TTAGGG)n in the karyotype of the species by Fluorescence *in situ* Hybridization. The dinucleotide motifs had a pattern of distribution clearly distinct from those of the tri- and tetranucleotides. The dinucleotide motifs are abundant and widely distributed in the chromosomes, located primarily in the subterminal regions. The tri- and tetranucleotides, by contrast, tend to be clustered, with signals being observed together in the secondary constriction of the homologs of pair 9, which are associated with the nucleolus organizer region. As expected, the (TTAGGG)n probe was hybridized in all the telomeres, with hybridization signals being detected in the interstitial regions of some chromosome pairs. We demonstrated the variation in the abundance and distribution of the different microsatellite motifs and revealed their non-random distribution in the karyotype of *P. rusticus*. These data contribute to understand the role of repetitive sequences in the karyotype diversification and evolution of this taxon.

## Introduction

The repetitive DNA sequences organized *in tandem* are abundant and widely distributed in the eukaryote genome (Charlesworth *et al.*, 1994). The microsatellite repeats, or Simple Sequence Repeats (SSRs), correspond to a class of repetitive DNA with less complex repetition units, composed of small, repeated *in tandem* motifs of one to six base pairs (Charlesworth *et al.*, 1994; Vieira *et al.*, 2016). These components of the genome are extremely useful as markers of genetic variation, due to hyper-polymorphism, and are used frequently in studies of population genetics. A number of mechanisms have been proposed to account for the high rates of variation found in the microsatellites, including the slippage of the DNA polymerase during replication and repair, the occurrence of unequal crossing-over, and ectopic recombination (Amos *et al.*, 2015).

Contradicting the assumption that microsatellites correspond to essentially neutral sequences, a number of studies have demonstrated their considerable density in the eukaryote genome and their conservation in many different lineages, which suggest a functional role for some sequences. Microsatellite motifs have been identified as modulators of transcription factors and chromatin structure, enhancers, and RNA regulators, as well as being considered preferential sites for meiotic recombination enzymes (for a review, see Bagshaw, 2017). Other studies have found evidence of their involvement in chromosomal rearrangements (Kamali *et al.*, 2011), and their tendency to accumulate in heteromorphic sex chromosomes indicates that they may participate in the differentiation and evolution of these chromosomes (Terencio *et al.*, 2013; Pucci *et al.*, 2016).

Microsatellite motifs are widely distributed in the genome, in both codifying and non-codifying regions, although some may have a non-random distribution, being organized in large genomic blocks, which can facilitate their detection in Fluorescence *in situ* Hybridization (FISH) experiments. The cluster organization pattern of these sequences in the karyotype may also favor recombination, either homologous or otherwise, which indicates the potential role of the sites as hotspots of chromosomal rearrangement, which is an important source of variation during karyotype diversification (Oliveira *et al.*, 2006; Armour, 2006; Vieira *et al.*, 2016).

A number of studies (Cuadrado and Jouve, 2007; Grandi and An, 2013; Ruiz-Ruano *et al.*, 2015) have reported associations between microsatellites and different classes of repetitive sequence (histone gene spacers, rDNA, and mobile genetic elements), as well as being a component of the heterochromatic blocks in the karyotype. Furthermore, the mapping of microsatellite motifs in the karyotype can help distinguish chromosome pairs, provide a better characterization of the different classes of heterochromatin, and contribute to the identification of chromosomal rearrangements, which means that they provide an extremely informative marker for the differentiation of karyotypes (Farré *et al.*, 2011; Paço *et al.*, 2013; Ruiz-Ruano *et al.*, 2016). However, few studies have adopted this approach up to now, in particular in amphibians (Peixoto *et al.*, 2015; 2016).

The monkey frog, *Pithecopus rusticus*, is an amphibian species endemic to the grasslands of the Araucaria Plateau, in the Atlantic Forest domain of southern Brazil (Bruschi *et al.*, 2014a). This species is currently known only from a small population found at the type locality, in the municipality of Água Doce, in the state of Santa Catarina, Brazil (Lucas *et al.*, 2010; Bruschi *et al.*, 2014a). The genus *Pithecopus* (Cope, 1866; recently resurrected from the genus *Phyllomedusa* by Duellman *et al.*, 2016) has 11 recognized species (Frost, 2019), all of which have highly conserved karyotypes, in terms of both the diploid number (2n=26) and chromosome morphology (Barth *et al.*, 2009; Bruschi *et al.*, 2012; Bruschi *et al.*, 2014b). The closest phylogenetically related species to *P. rusticus* are *P. ayeaye*, *P. megacephalus*, *P. centralis*, and *P. oreades* (Bruschi *et al.*, 2014a), which are all found on the plateaus and highland areas of the Cerrado savannas of central Brazil (Faivovich *et al.*, 2010; Bruschi *et al.*, 2014a).

Cytogenetic data on *P. rusticus* will be fundamental to a better understanding of the origin and diversification of this taxon, given its restricted geographic distribution, which is completely disjunct from those of other species of the genus. In this study, we present the genomic organization of seven microsatellite motifs and the (TTAGGG)n repeats in the karyotype of *P. rusticus*, and we demonstrate the non-random distribution of these repeats, in association with the 45S rDNA gene.

## Material and Methods

### Biological samples

Tissue samples were obtained from 6 males specimens of *Pithecopus rusticus* paratypes collected during the fieldwork, between 2009 and 2012, that led to the original description of the species (Lucas *et al.*, 2010; Bruschi *et al.*, 2014a). Vouchers are deposited in Coleção de Anfíbios da Universidade Comunitária da Região de Chapecó (UNOCHAPECÓ), Santa Catarina States, Brazil under numbers CAUC0763, CAUC13356, CAUC0766, CAUC0768, CAUC0770 and CAUC0771. These specimens were collected at the type locality, in the municipality of Água Doce, in the state of Santa Catarina, in southern Brazil (26º35’59.90” S, 51º34’39.40” W). The cell suspensions were prepared from intestinal and testicular tissue (Bruschi *et al.*, 2014a), which had been treated with 2% colchicine, using procedures modified from King and Rofe (1976) and Schmid (1978). The cell suspensions were dripped onto clean microscope slides and stored at -20°C. The nucleolar organizer regions (NOR) were revealed by Ag-NOR technique (Howell and Black, 1980) and cofirmed by *in situ* hybridization with 28S rDNA probes, isolated, cloned and sequenced according to Bruschi *et al.* (2012) from *Pithecopus hypochondrialis*.

### Probes of the microsatellite repeats and telomeric (TTAGGG)n sequences

The microsatellites were analyzed using oligonucleotide probes – CA_(15)_, GA_(15),_ GAA_(10)_, CAG_(10)_, CGC_(10)_, GACA_(4)_, GATA_(8)_ – marked directly with Cy5 fluorochrome (Sigma Aldrich) at the 5’ end during the synthesis of the DNA. The telomeric (TTAGGG)n repeats were produced by PCR amplification using telomeric primers F (5’ TTAGGG 3’) and R (5’ CCCTAA 3’), with the product of this amplification being marked directly by the incorporation of 11-digoxigenin-dUTP, following the protocol described by Guerra (2012).

### Fluorescence *in situ* Hybridization (FISH) experiments

The microsatellite FISH experiments were based on the protocol propose by Kubat *et al.* (2008). For telomeric repeats, the hybridizations were conducted according to the protocol of Traut *et al.* (2001), with the following modifications: the slides were washed in 0.2N HCl for 2 minutes, followed by two washes in PBST for 3 minutes, with the chromatin structure being stabilized in 1%/150 mM PBS 1X formaldehyde, for 10 minutes, then washed again in PBST for 3 minutes, and dehydrated in an increasing alcohol series (at 70%, 80%, and 96%) for 3 minutes. The samples were denatured in deionized 70%/2xSSC formamide for 3 minutes at 70°C and then dehydrated again in an increasing alcohol series (at 70%, 80%, and 96%).

For hybridization, each slide received a final concentration of 50ng/uL of the probe. After 24 hours of hybridization in a wet chamber at 37°C, the slides were washed in 2X SSC at 42°C and in PBST for 5 minutes, and then dehydrated again in an increasing alcohol series (at 70%, 80%, and 96%) for 3 minutes. The slides were then incubated in NFDM buffer for 15 minutes, and the signal was detected using the antidigoxigenin antibody in NFDM buffer for 1 hour in a wet and dark chamber, at room temperature. The slides were then washed again, three times, in 0.5%/4xSSC Tween for 5 minutes, dehydrated in the alcohol series, and counterstained with DAPI. Ten metaphases per individual were photographed under Olympus BX-51 epifluorescence microscope.

## Results

**Figure 1 f1:**
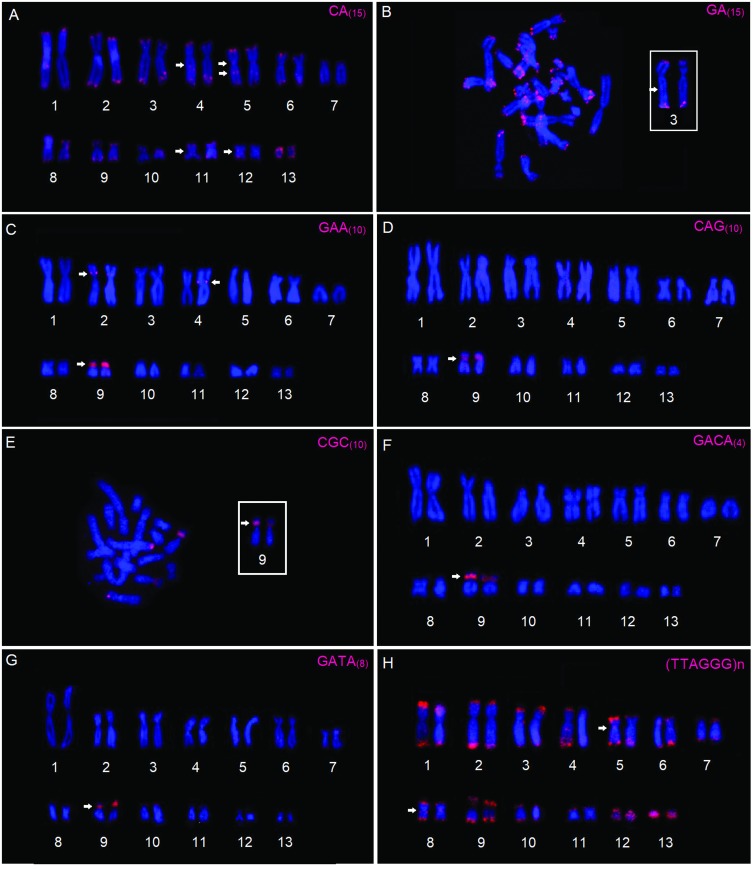
Metaphase chromosomes of *Pithecopus rusticus* (2n=26) submitted to Fluorescence *in situ* Hybridization (FISH) with the microsatellite for the repeats of (A) CA_(15);_ (B) GA_(15);_ (C) GAA_(10);_ (D) CAG_(10);_ (E) CGC_(10);_ (F) GACA_(4);_ (G) GATA_(8),_ and (H) the telomeric (TTAGGG)n repeats. The partial karyotypes are presented in (B) and (E). The arrows indicate the interstitial and pericentromeric signals. In (B) and (E), the chromosome pairs with GA_(15)_ and CGC_(10)_ signals (respectively) are shown in the boxes.

The diploid number in all specimens analyzed showed 26 chromosomes. The dinucleotide microsatellite probes CA_(15)_ (Figure 1A) and GA_(15)_ (Figure 1B) were distributed abundantly in all the chromosomes and presented signals of hybridization in the subterminal regions. Interstitial CA_(15)_ hybridizations were also observed in the long arms of the homologs of pairs 4 and 5, and in the pericentromeric regions of the short arms of pair 5 and the long arms of pairs 11 and 12 (Figure 1A). Interstitial hybridizations of the GA_(15)_ were detected in the long arm of the homologs of pair 3 (Figure 1B).

**Figure 2 f2:**
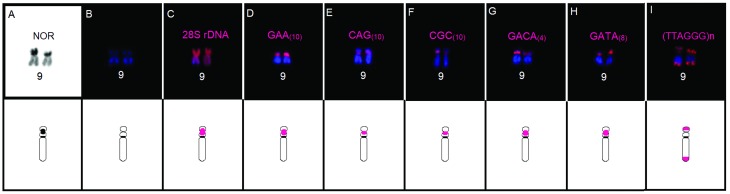
Homologs of chromosome pair 9 in *Pithecopus rusticus* and diagrams of the co-location of the hybridization signals highlighted in (A) the Nucleolus Organizer Region (Ag-NOR); (B) secondary constrictions (DAPI); (C) the 28S rDNA; (D–H) distribution patterns of different microsatellite markers; (I) telomeric (TTAGGG)n repeats.

The trinucleotide – GAA_(10)_, CAG_(10)_, CGC_(10)_ (Figure 1C-E) – and tetranucleotide – GACA_(4)_ and GATA_(8)_ (Figure 1F-G) – microsatellites presented clustered hybridization signals in the secondary constrictions of the homologs of pair 9, involving the secondary constriction related to the NOR site described (Bruschi *et al.*, 2014a; present study – Figure 2A-C). Considerable variation in signal strength was also observed for each marker, with GAA_(10)_, GACA_(4)_ and GATA_(8)_ presenting stronger signals (Figure 2). Interstitial signals of GAA_(10)_ (Figure 1C) were also detected on the short arms of pair 2 and in one of the homologs of pair 4.

The *in situ* hybridization detected (TTAGGG)n sequences in all the chromosomes of the *P. rusticus* karyotype (Figure 1H)*.* Hybridization signals were also detected in the pericentromeric region of pairs 5 and 8. Intense hybridization signals of (TTAGGG)n sequences were detected in the homologs of pair 13 (Figure 1H) [Fig f2].

## Discussion

The *in situ* mapping of the different microsatellite repeats contributed to the understanding of the chromosomal organization of this repetitive DNA in the karyotype of *Pithecopus rusticus*. The results of the present study indicated that the dinucleotide motifs has a chromosomal distribution pattern distinct from those of tri- and tetranucleotides. The CA_(15)_ and GA_(15)_ microsatellites are abundant and widely distributed in the chromosomes, and are located primarily in the subterminal regions of the chromosomes.

Repeats of (CA)n and (GA)n appear to be the most common microsatellite dinucleotide motifs in animal genomes (Ruiz-Ruano *et al.*, 2015) and have been linked to the high rates of recombination observed in these organisms (Guo *et al.*, 2009), due to their affinity with the recombination enzymes (Biet *et al.*, 1999). The distribution of these motifs, especially in the subterminal region, may also be important for the stabilization of the chromosomes terminal portions. A similar accumulation of dinucleotide repetitions in the chromosomes subterminal regions has been observed in some species of amphibians of the genus *Ololygon* (Peixoto *et al.*, 2015, 2016), in several species of fish (Poltronieri *et al.*, 2013; Schneider *et al.*, 2015; Pucci *et al.*, 2016), grasshoppers (Ruiz-Ruano *et al.*, 2015), and plants (Vanzela *et al.*, 2002; Torres *et al.*, 2011). The arrangement of repetitive DNA in the subtelomeric region appears to be a common characteristic of the eukaryotic chromosome, driven by different mechanisms of enrichment (transposable elements, satellites and microsatellites), which have played a fundamental role in the formation of the heterochromatin in these regions (Torres *et al.*, 2011). In a study of fission yeasts, Tashiro *et al.* (2017) confirmed the importance of this type of subterminal region organization for telomere function, regulation of adjacent genes and chromosome homeostasis.

By contrast, the tri- and tetranucleotide motifs mapped here presented a clustered distribution in the same chromosomal region, as observed in the pericentromeric region, extending to the interstitial portion of the homologs of pair 9. The patterns of genomic organization (dispersed or clustered) of repetitive sequences likely reflect distinct evolutionary events (Ruiz-Ruano *et al.*, 2015; Utsunomia *et al.*, 2018) and the potential of each motif for expansion (Pokorná *et al.*, 2011; Kejnovský *et al.*, 2013). Several studies have shown that the accumulation of microsatellites in the eukaryotic genomes is not random, and closely-related species tend to present a tendency for accumulation of repetitions in a specific chromosome (Cuadrado and Jouve, 2007; Ruiz-Ruano *et al.*, 2015; Zheng *et al.*, 2016; Utsunomia *et al.*, 2018), which may reflect an important functional role (Cuadrado and Jouve, 2007; Ruiz-Ruano *et al.*, 2015).

*Pithecopus rusticus* has a single NOR site located in the subterminal region of chromosomal pair 9 (Bruschi *et al.*, 2014a; presente study), in which hybridization signals were detected of both trinucleotide [GAA_(10)_, CAG_(10)_, CGC_(10)_] and tetranucleotide [GACA_(4)_ and GATA_(8)_] repeats. For example, the distribution of the (GAA)n sequence was related to chromosomal rearrangements/modifications involving primarily NOR-bearing chromosomes, as observed in a number of different lineages of wheat, *Triticum* spp*.* (Adonina *et al.*, 2015). The frequent association between microsatellite repeats and the NORs is not entirely unexpected, given that the massive presence of microsatellite repeats has been observed in intergenic spacers (IGSs) in the rDNA (Ruiz-Ruano *et al.*, 2015; Agrawal and Ganley, 2018). The association between microsatellite repeats and IGS regions, in particular di- and trinucleotide motifs has been confirmed by analysis of reads combined with FISH experiments in grasshoppers (Ruiz-Ruano *et al.*, 2015) and also corroborated in the present study.

While the centromere is formed primarily of repetitive DNA, none of the microsatellite repeats were detected in this region in *P. rusticus*, which may be related to the fact that the centromeres reduce recombination rates and, as a consequence the amplification of these microsatellite motifs in this region (Guo *et al.*, 2009). Therefore, the microsatellite sequences are normally found in the regions adjacent to the centromere, as observed in the pericentromeric signals of the CA_(15)_ repeats in some of the *P. rusticus* chromosomes.

As expected, the (TTAGGG)n probes hybridized in all terminal regions of the chromosomes, since this sequence is highly conserved in all vertebrates (Bolzán, 2017). In addition to these signals, our FISH experiments revealed large blocks of (TTAGGG)n repeats distributed in the internal regions of the chromosomes, that is, Interstitial Telomeric Sequences (ITSs). The mapping of ITSs seems to be useful for the detection of interchromosomal rearrangements, such as fusions, or intrachromosomal rearrangements of the inversion type (Teixeira *et al.*, 2016; Bolzán, 2017). However, the ITSs are also capable of spreading rapidly, as observed in the pericentromeric regions, probably independently (Wiley *et al.*, 1992; Rovatsos *et al.*, 2011; Bruschi *et al.*, 2014b).

The detection of ITSs in *P. rusticus*, in addition to the presence of interstitial signals in the karyotypes of the other species of the family Phyllomedusidae analyzed to date (Gruber *et al.*, 2013; Barth *et al.*, 2014; Bruschi *et al.*, 2014b), indicates that the presence of this type of sequence is recurrent in these frogs. The intrachromosomal variation in the telomeric repeats found in different *Phyllomedusa* species (e.g., *Phyllomedusa vaillantii*, *Phyllomedusa tarsius*, *Phyllomedusa distincta*, and *Phyllomedusa bahiana*) reflects different patterns of (TTAGGG)n signals in the interstitial regions of the chromosomes of these species (Bruschi *et al.*, 2014b). However, the clear conservation of the chromosome structure in this group, the origin of the ITSs detected in the present study probably cannot be explained by rearrangements, but may be a result of the amplification of (TTAGGG)n repeats, which occurred independently during the chromosomal evolution of these species. Interestingly, these ITSs are associated with heterochromatin, given that they were detected in pericentromeric regions, coinciding with the C-band positive blocks reported by Bruschi *et al.* (2014a), and a similar pattern has been observed in the *Phyllomedusa* species (Bruschi *et al.*, 2014b), and in other anuran species (Schmid and Steinlein, 2016). As observed in *P. rusticus*, intense hybridization signals were also detected in the homologs of pair 13 in *Phyllomedusa vaillantii*, indicating that the (TTAGGG)n sequence is an important component of the repetitive DNA of these chromosomes in the phyllomedusids (Bruschi *et al.*, 2014b).

A few studies have investigated the cytogenetic characteristics of the phyllomedusids, including descriptions of karyotypes, the identification of heterochromatic regions and NOR sites (Morand and Hernando, 1997; Barth *et al.*, 2009, 2013, 2014; Paiva *et al.*, 2010; Bruschi *et al.*, 2012, 2013, 2014a, 2014b; Gruber *et al.*, 2013). *Pithecopus rusticus* is apparently limited to a small and isolated population, the evaluation of the composition and distribution of repetitive DNA in the genome is fundamental to understand the role of these sequences in the evolution of the karyotype of this taxon. DNA sequences that are widely repeated in the genome are capable of evolving independently and also serve as a substrate for recombinations and chromosomal rearrangements (Kamali *et al.*, 2011; Carmona *et al.*, 2013; Utsunomia *et al.*, 2018) and in small and interbreeding populations, such as *P. rusticus*, evolutionary novelties may arise frequently and will be fixed rapidly in the population (Gemayel *et al.*, 2010). Therefore, the results of the present study provide important insights into the diversification and distribution of repetitive sequences in the *P. rusticus* karyotype, which may be useful, in particular, for comparative analyses, and the understanding of evolutionary mechanisms that determine the characteristics of this taxon, in addition to the molecular cytogenetics of amphibians, in general.
